# Frontal Fibrosing Alopecia: A Review

**DOI:** 10.3390/jcm10091805

**Published:** 2021-04-21

**Authors:** María Librada Porriño-Bustamante, María Antonia Fernández-Pugnaire, Salvador Arias-Santiago

**Affiliations:** 1 Dermatology Department, University Hospital La Zarzuela, 28023 Madrid, Spain; 2 Dermatology Department, University of Granada, 18016 Granada, Spain; 3 Dermatology Department, University Hospital San Cecilio, 18016 Granada, Spain; marian.fer@telefonica.net; 4 Dermatology Department, University Hospital Virgen de las Nieves, 18014 Granada, Spain; salvadorarias@hotmail.es; 5 Institute of Biosanitary Investigation IBS, School of Medicine, Granada University, 18016 Granada, Spain

**Keywords:** frontal fibrosing alopecia, scarring alopecia, bulge, trichoscopy, histopathology, treatment

## Abstract

Frontal fibrosing alopecia is a scarring alopecia, the prevalence of which is increasing worldwide since its first description in 1994. The reason for this emerging epidemic may be a higher exposure to an unknown trigger, although its aethiology and pathogenesis still remain enigmatic. Clinical, trichoscopic, sonographic, and histopathologic findings are allowing clinicians to understand more aspects about this type of cicatricial alopecia. Several treatments have been used in frontal fibrosing alopecia, although the 5-alpha reductase inhibitors seem to be the most promising. The aim of this report is to provide a compilation about the published data regarding frontal fibrosing alopecia in a narrative review.

## 1. Introduction

### 1.1. Definition and History

Frontal fibrosing alopecia (FFA) was described in 1994 by Kossard as a progressive scarring alopecia in postmenopausal women, affecting the frontal and temporoparietal hairline, and was initially called postmenopausal frontal fibrosing alopecia [1]. FFA was referred to in 1997 by Kossard as a frontal uncommon variant of LPP [2]. However, this is still flatly controversial, and other authors consider that FFA is a distinct entity from LPP [3]. Nowadays, FFA is probably one of the most common types of scarring alopecia, if not the most common [4]. The gradual increase in publications related to FFA may be due to a higher awareness among clinicians in regard to this alopecia [5]. However, a higher prevalence of a still unknown trigger in recent years may be another relevant factor in this epidemic of FFA.

### 1.2. Aim and Methods

The aim of this report is to perform an updated and complete review about FFA regarding epidemiology, aetiopathogenesis, clinical characteristics (clinical description, trichoscopy, image techniques), prognostic factors, histopathology, diagnosis, differential diagnosis, and treatment. For that, an exhaustive review of all of the references related to FFA and published in PubMed has been done by searching for “frontal fibrosing alopecia”, including references written in English, Spanish, German, and French, from 1994 to 2021. A total of 487 articles have been reviewed. Articles with a more significant number of patients have been included. In addition, publications with a lower number of patients that provided new information about FFA have also been incorporated.

## 2. Epidemiology and Demographic Data

There are no specific data about the worldwide prevalence of FFA so far. Recently, the overall crude prevalence for FFA in New York City has been estimated at about 0.015% [6].

FFA was described initially as affecting almost exclusively postmenopausal women. However, although this group seems to be the most frequently affected, it is not the only one. The first report of a man with FFA dates from 2002 [7], but many more have been published since then [3,8,9]. Moreover, a fair number of cases of FFA in premenopausal women have been published since its first description [10,11]. In spite of this, FFA begins at postmenopausal age in around 83% to 95% of women (Caucasians and Asians) [3,12,13,14,15]. Nevertheless, the biggest study of FFA in black-skinned patients reported that 74% of the women were premenopausal [16]. Regardless, it seems that premenopausal cases are also increasing.

The mean age of onset of FFA ranges from 56 to 63 years [3,12,17]. Even so, some cases have been published about younger patients, the youngest one being a 15-year-old female [18]. In a 355 patient cohort, the rate of early menopause (14%) was higher than in the general population (6%); moreover, 13% of patients had undergone a hysterectomy [3]. The mean time to the diagnosis reported in different studies is about 3.4 to 5.3 years [3,13,19]. Assessing the exact duration of the disease can be difficult sometimes, because its slow progression makes it complicated for the patient to detect the real time of onset.

Male patients with FFA seem to be affected with FFA at a younger age than women, with a mean age of onset of 47.3 years [20]. FFA in men is probably underdiagnosed because of its overlap with androgenetic alopecia (AGA); indeed, the main complaint among men having FFA is usually eyebrow loss rather than scalp alopecia [9]. 

Regarding the human race, FFA has been described worldwide, although most cases have been reported in European and North American countries, mainly among Caucasians and fewer among black-skinned populations [16], whereas only a few cases have been reported in Asia, where the incidence may be lower [21,22,23,24]. Indeed, a recent study regarding the prevalence of FFA and LPP in New York City observed that the prevalence of the combined group LPP/FFA was highest among non-Hispanic Caucasians (0.091%) [6].

With reference to external factors, a study found that patients with FFA belonged to a more affluent group compared to both a comparable control group with other types of alopecia and to a general control group [13].

## 3. Aetiopathogenesis

**Figure 1:**
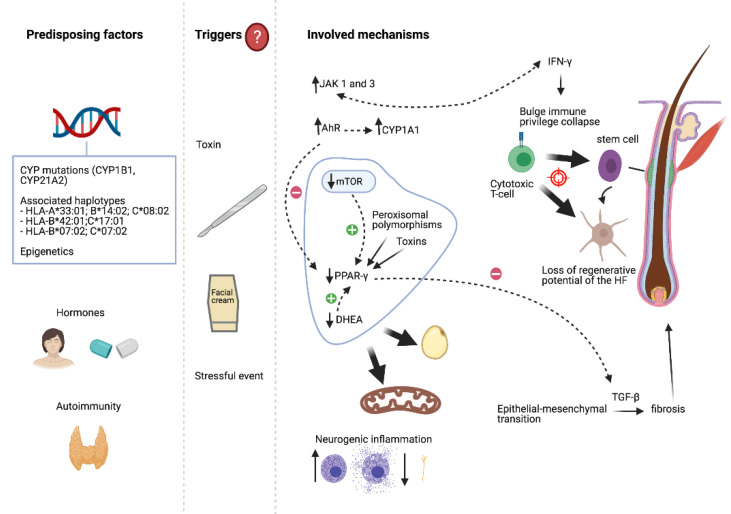
Different unknown triggers, such as facial creams/sunscreens, environmental toxins, surgery, or a stressful situation, may lead to FFA in genetically susceptible individuals. Autoimmunity may also contribute to the predisposition, so people with one autoimmune disease are more likely to have another one. Hormonal factors probably play a role in the development of FFA. Discontinuous lines indicate a regular relationship between two elements. JAK: Janus kinase; AhR: aryl hydrocarbon receptor-kynurenine; mTOR: mammalian target of rapamycin; PPAR-γ: peroxisome proliferator-activated receptor γ; DHEA: dehydroepiandrosterone; HF: hair follicle; IFN: interferon; TGF: transforming growth factor; HLA: human leukocyte antigen; CYP: cytochrome. Created with BioRender.com (accessed on 1 March 2021).

The aetiopathogenesis of FFA remains unknown, although hormonal factors, autoimmunity, genetic susceptibility, and some exogen factors are thought to play a role (Figure 1) [14].

The loss of the immune privilege of the hair follicle would be the starting point in the development of scarring alopecias [25]. This bulge immune privilege collapse may be induced by IFN-γ [25]. In FFA, a Th1-biased cytotoxic T cell autoimmune reaction against the hair follicle in the infundibular region and, to a more variable degree, the isthmic region, seems to play a major part [26]. This damage would include the bulge area, where stem cells are placed, leading to a loss of the regenerative potential of the hair follicle and its total destruction. A decrease to the absence of labelling with Ki-67, a proliferative marker, and a downregulation of the hair follicle epithelial progenitor cell marker keratin 15, within the bulge area, have already been described in LPP [27,28]. The melanocyte of the hair follicle might be an antigenic target in FFA [29]; this is supported by the lower melanocyte count found in the upper follicle in lesional skin from FFA patients (not seen in LPP) [26,30]. 

The onset and progression of FFA in a woman with psoriasis, treated with ustekinumab (an anti-IL12/23 p40 monoclonal antibody), suggests that the Th1 and Th17 pathways do not play a major role in FFA [31]. 

On the one hand, in LPP, the downregulation and the abnormal function of the peroxisome proliferator-activated receptor γ (PPAR-γ) have been proposed as the initial triggers of the inflammation. PPAR-γ plays a central role in lipid homeostasis and in the differentiation and maturation of sebocytes [28]. Targeted deletion of PPAR-γ in the follicular stem cells of the bulge in mice causes a phenotype resembling scarring alopecia, suggesting that this receptor is essential for healthy pilosebaceous units [32]. Peroxisomal polymorphisms and/or environmental triggers (toxins such as dioxin) may lead to this localized and acquired PPAR-γ dysfunction [32].

The mammalian target of rapamycin (mTOR) is a pathway that combines signals and acts as a central regulator for metabolism, growth and cell proliferation, and is a major regulator of adiposity due to PPAR-γ activation [33]. A recent study has found that the expression of all mTOR signaling pathway proteins are decreased in the lesional epidermis of patients with LPP/FFA [34]. In addition, dehydroepiandrosterone (DHEA) has an immunomodulatory role, and is essential for the stimulation of PPAR in the transcription of genes, fat metabolism, and in mitochondrial activity [35]. It is possible that the benefits obtained by the treatment of FFA with 5a-reductase inhibitors are the result of DHEA impediment in reaching its final conversion to dihydrotestosterone [35].

Moreover, transmission electron microscopy and global metabolomics profiling data have identified defects in mitochondrial β oxidation of fatty acids, leading to the accumulation of medium- and long-chain fatty acids, along with decreased levels of (antioxidant) glutathione and elevated levels of oxidized glutathione (a marker of oxidative stress) in both lesional and non-lesional FFA scalp samples. Therefore, mitochondrial dysfunction may be an early process in the pathogenesis of FFA [36].

The expression of Snail1 noted in the fibrotic dermis in FFA suggests that the fibroblasts are, in part, derived from the hair follicle via an epithelial-mesenchymal transition process [23]. The transforming growth factor-β (TGF-β) is an inducer of this transition, promoting fibrosis, differentiating epithelial cells and quiescent fibroblasts intro myofibroblasts, and increasing the expression of the extracellular matrix [23]. In this way, increased expression of the Treg marker FOXp3+ has been described in LPP/FFA, and the Treg-mediated TGF-β-signaling appears to drive fibrosis through this transition [37]. Moreover, PPAR-γ is a negative regulator of fibrotic events induced by TGF-β1 [38]. 

On the other hand, the expression of Janus kinase (JAK) 1 and 3 are significantly upregulated in dermal inflammatory cells in patients with LPP [39]. Therefore, JAK inhibition may reduce IFN-mediated inflammation associated with LPP and prevent further hair follicle destruction.

Neurogenic inflammation is another hypothesis for the pathogenesis of scarring alopecias [40]. The total number of mast cells, along with the proportion of degranulating ones, are increased in the perifollicular bulge region in LPP/FFA [41]. Moreover, decreased epidermal nerve fiber density has been found in FFA, as well as an increased concentration of substance P in the unaffected areas compared to the affected ones, and higher expression of cGRP in affected areas of cases with mild inflammation [42].

A new hypothesis proposes that FFA may arise as a result of excessive facial photo-protection, with a resultant disturbance in immunological homeostasis mediated via the aryl hydrocarbon receptor-kynurenine pathway axis (AhR/KP), leading to the collapse of immune privilege at the hair bulge [43]. Overexpression of the aryl hydrocarbon receptor (AhR) in the epidermis of FFA and LPP has been newly described in both unaffected and affected scalp [44]. CYP1A1 gene, the expression of which is directly controlled by AhR signaling to metabolize xenobiotics, has been described as being upregulated in affected and unaffected skin in LPP [32]. Multiple chemical substances can inhibit the metabolism of CYP enzymes and thereby indirectly cause AhR activation. Moreover, AhR is involved in the suppression of PPAR-γ [44]. 

A new study has identified the presence of circulating microRNAs as being highly predictive of disease status in FFA [45].

### 3.1. Hormones

Hormonal factors are thought to play a role in FFA, due to their higher frequency in women, especially postmenopausal ones, and their response to 5-alpha reductase inhibitors [5].

Estrogens produce a decrease in the hair shaft growth and favor the catagen to telogen transition [46]. Therefore, the decrease of estrogens due to physiological or surgical menopause could alter the control of the hair cycle and be the trigger for the inflammatory attack on the hair follicle in susceptible patients [3]. Moreover, the potential role of estrogens as an anti-fibrotic and immunomodulatory agent in FFA has been discussed [47].

In postmenopausal women, the course of the disease appears to be unaltered when hormone replacement therapy is introduced, and it does not seem to prevent the onset of the disease either [2,5]. However, early menopause could be an issue involved in the premature development of FFA, or may imply a higher risk of developing FFA [2,14,48]. 

The use of an intrauterine device as a contraceptive may protect against the development of FFA, whereas the intake of tamoxifen with the induction of a low-estrogen environment around the hair follicle may trigger or maintain the pathogenic process of FFA [48].

A few male patients have been reported as having FFA and a history of prostate cancer treated with neoadjuvant hormonal therapy (antiandrogens or estrogens) before the onset of FFA [49,50]. One man was receiving testosterone because of an iatrogenic hypogonatotropic hypogonadism and developed FFA afterwards; moreover, he developed alopecia earlier than his brother, who also had FFA [9].

Serum hormonal levels are not consistently altered in women diagnosed with FFA, although this does not exclude a potential hormonal involvement by a local mechanism [51]. In fact, a recent study about hormonal dysfunction found that LPP is associated with androgen excess (testosterone or DHEAS), whereas FFA is related to androgen deficiency (32.1% of patients) [52]. According to these findings, DHEAS and androstendione have been found to be lower in women with FFA compared to a control group [19,53]. Moreover, abnormal estrogen and testosterone values have been associated with lesser disease activity [18]. Serum levels of the follicle-stimulating hormone (FSH) in premenopausal women with FFA have been demonstrated to be lower (although within normal ranges) compared to a control group [54]. In the same study, the levels of luteinizing hormone (LH) and FSH were significantly lower in postmenopausal women with premenopausal onset in comparison to the ones with postmenopausal onset. Progesterone serum levels have also been noted to be lower in patients having both FFA and rosacea [19]. 

### 3.2. Associated Diseases and Autoimmunity

About 9.7% to 30% FFA patients, mostly women, have an associated immune disorder [13,55]; the most frequent are thyroid diseases, especially hypothyroidism (8–44.6%) [3,13,14,56]. Other autoimmune conditions, which have been described together with FFA, are referred to in Table 1 [3,13,14,56,57,58,59,60]. Several studies have demonstrated that patients with FFA are significantly more likely to have systemic lupus erythematosus, while patients with LPP and FFA are less likely to have diabetes [61,62]. Vitiligo and FFA are sometimes associated, and both diseases may share common pathogenic pathways [63].

Only one case of a woman with common variable immunodeficiency (CVID) and FFA has been published [64]. 

Different forms of lichen planus, such as oral [3,14,65], vulvar and conjuntival [14,66], nail [67], and cutaneous [3,10,14] lichen planus, have been described together with FFA. However, LPP appears along with cutaneous or mucous lichen planus in up to 50% of patients, whereas around 2 to 18% of patients with FFA have lesions of lichen planus in other locations [10,12,13,56]. Actually, the clinical form of lichen planus most commonly linked to FFA is LPP, in 0.8 to 25% of patients [2,3,11,13,17,68]. Vulvar lichen sclerosus [13,56,69] and lichen planus pigmentosus (LPPigm) [70] have also been found concurrently with FFA. One case of actinic lichen planus triggered by drug photosensitivity and preceding the onset of the FFA has been published [71]. Another entity placed in the spectrum of LPP, Graham-Little-Piccardi-Lasseur Syndrome (GLPLS), consisting of keratotic papules on the limbs or trunk, multifocal cicatricial alopecia, and non-atrophic axillary and pubic hair loss, has also been described concomitantly with FFA [72]. 

A high prevalence of atopy (43.9%) has been demonstrated in a FFA patients cohort [56]. However, according to different studies, one of the most frequently associated cutaneous condition seems to be rosacea, with a prevalence of 15 to 61% [19,55,56]. Interestingly, patients with more severe FFA appear to be more likely to have rosacea than those with mild grades of alopecia [19].

Androgenetic alopecia (AGA) is observed concomitantly with FFA in 16 to 57% of women [3,11,12,19,68,73] and in 67 to 83% of men [3,74]. An overlap of FFA, AGA, and frontal fibrosing in a pattern distribution (FAPD) has also been described [73]. One case of a patient having AGA, FFA, and trichotemnomania has also been published [75].

A woman with continued hair growth in a vascular nevus in an area otherwise affected by FFA has been recently described; the Renbök phenomenon describes how the emergence of one skin condition inhibits another [76].

### 3.3. Genetic Factors

Human leukocyte antigen (HLA)-DR1 has been related to familial cases of LPP and LGLPs [77]. Later, an association of HLA-B7 in familial cases of LPP (but not in sporadic cases) was published [78]. Since the first report of familial FFA in 2010 [79], more cases have been published, the largest series so far being one including 20 cases from nine different families [80]. A subsequent report regarding two sisters with FFA found negativity for HLA-DR1 [81]. A woman with FFA and her daughter with LPP, both with the same HLA type (DRB1*04,13; DQB1*03:02,06), have also been reported [82], as well as a woman with FFA whose mother had FAPD [83].

A study including 13 cases of familial FFA found that most of the patients of that cohort shared HLA-A*33:01; B*14:02; C*08:02, suggesting that this haplotype may predispose to familial FFA [84]. Moreover, it was found to be linked with the CYP21A2 gene p.V281L mutation (from congenital adrenal hyperplasia). HLA-B*07:02 was also included in the haplotypes of some of the patients.

A recent GWAS demonstrated a significant association with FFA in four genomic loci: 2p22.2, 6p21.1, 8q24.44, and 15q2.1. Fine mapping within the 2p22.2 and 6p21.1 loci revealed associations with a presumed casual missense variant in CYP1B1 (which encodes a member of the cytochrome P450 family involved in the oxidative metabolism of estrogens) and the HLA-B*07:02 allele, respectively [85]. 

A study of HLA profiles in a familial cluster (seven members with FFA and four unaffected) and seven sporadic cases found two susceptibility haplotypes in the familial cases (C*17:01:01:02/B*42:01:01:01 and C*07:02:01:03/B*07:02:01:01), which were also shared by three unaffected family members [86]. This suggests that other genetic or environmental factors may modulate the HLA association. Moreover, five out of seven sporadic cases also shared the referred haplotypes (3/7 the former, 2/7 the latter). Another woman with sporadic FFA presented HLA-A*33:01:01; B*14:02:01; C*08:02:01, which was previously associated to FFA [84]. 

A review of eight cases of familial FFA from four different families (mother and daughter) revealed that all of the mothers were postmenopausal at the time of the diagnosis and had an advanced alopecia, whereas all of the daughters were premenopausal and had a mild form of the disease [87]. This has been confirmed in subsequent familial studies [80].

The occurrence of the disease in families can indicate exposure to a common environmental trigger, probably enhanced by a genetic predisposition. The possibility of a common environmental exposure is supported by a case of connubial FFA in a genetically unrelated couple, although it may have been coincidental [88].

An autosomal dominant transmission with reduced penetrance has been proposed as a hypothetic inheritance pattern in FFA [89]. Three monocigotic twins have been identified with FFA, which reinforces the fact that epigenetics may play a fundamental role in FFA pathogenesis, in addition to a genetic predisposition [47].

With regards to a family history of the disease, this is reported by 5 to 8% of patients with FFA [3,16].

### 3.4. Surgical Procedures and Hair and Skin Care Products

Some cases of FFA or LPP developed after hair transplantation for AGA, so hair transplants may also be affected [8,90,91]. In addition, one female developed FFA after face lift surgery [91]. Explanations for these situations include the Koebner phenomenon induced by surgical trauma, or an autoimmune attack from a follicle antigen liberated during surgery or induced by a post-surgery pro-inflammatory environment [91]. In that way, the Koebner phenomenon may have also been the cause in a woman with FFA who developed LPP in the areas of wig attachments [92].

The use of sunscreens has been proposed as a possible trigger for the development of FFA since the publication of a study that found a higher use of these products in FFA patients compared to a control group [93]. This finding has been confirmed in most subsequent reports [94,95], although there are also some FFA patients who had not used sunscreens and yet still developed FFA [74]. There is an interesting case of a woman with FFA who had hair regrowth following the cessation of sunscreen use on the forehead [96]. However, daily facial sunscreen application has not been associated with worsening disease progression in treated FFA patients [97]. Interestingly, in a random review of hair care products, 60% of leave-on hair products and 51% of wash-off products contained a chemical sunscreen [98]. 

Nanoparticles of titanium dioxide (a substance found in physical sunscreens) have been detected along the hair shafts of a patient presenting FFA [99]. It is not clear if titanium dioxide can penetrate the stratum corneum, but it is known that it can deposit itself in the follicular opening. However, these deposits on hair shafts have not been found in subsequent studies [100], or have been observed in both patients and control subjects [101], hence this is meaningless. Furthermore, most of the sunscreens used by patients with FFA are chemical ones [95].

A higher frequency of positive patch test in FFA patients, mainly for fragrances [93], benzyl salicylate [102], cobalt, nickel, and potassium dichromate [103], have been found in different studies, although others do not suggest any association of FFA with photoallergy to several cosmetic-related substances (including chemical sunscreen filters) or titanium dioxide [100,104]. Moreover, the positive patch test may be a consequence of increased exposure to these substances.

Therefore, the relation of sunscreen use and FFA remains flatly controversial [105]. Concordant results across multiple population-based studies suggest that a true correlation between sunscreen use and FFA may exist, but this does not necessarily imply causation [105]. The higher usage may simply reflect a new behavior adopted because of the alopecia, or may even reflect higher economic status [106]. Moreover, the increasing number of FFA cases reported in black-skinned patients, among whom the rates of sunscreen use are generally low, is also less consistent with the sunscreen hypothesis of causality [16,105]. 

Curiously, the frequency of shampooing has been found to be significantly lower in patients with FFA compared to a control group; this may reflect the common concern that hair washing may worsen hair loss, or suggest the possibility that frequent shampooing reduces the risk of developing FFA by achieving a more efficient removal of exogenous particles that could penetrate the follicular infundibulum and trigger the inflammatory response in patients who are genetically predisposed [93,107].

### 3.5. Drugs, Medications, and Other Factors

Some studies have reported a preponderance of non-smokers within FFA patients [3,13] or a less severe FFA, also after smoking cessation [108,109]. However, the issue about smoking and FFA is still controversial, and it is not supported by other studies [93]. 

With regard to specific medications, no clear association was found in connection with the onset of FFA [13]. However, a higher history of oral contraceptive in a control group compared to FFA patients has been found [93], although it may be related to a possible selection bias in the control group (hospital staff) [106].

Recently, the case of a woman who received nilotinib (a tyrosine kinase inhibitor) for chronic myeloid leukemia and developed keratosis pilaris, body hair loss, eyebrow alopecia, and frontal hairline recession has been published [110].

Regarding dietary habits, a greater consumption of buckwheat and millet groats has been reported in FFA patients [111], although subsequent studies have not found any association between phyto-estrogens (i.e., soy) or natural PPAR-γ agonists (i.e., grapes) and FFA [95].

Moreover, a relationship between occupational exposure to alkylphenolic compounds in women with FFA has been described; these substances have been shown to interact with PPAR-γ and inhibit transformation of DHEA to DHEAS [95].

The occurrence of an intense and stressful event just before the onset of FFA has been referred to by some patients (76%) [13,80,107].

## 4. Clinical Characteristics

### 4.1. Clinical Features

FFA is a scarring alopecia characterized by frontal and temporoparietal hairline recession, leading to a cicatricial band, which tends to contrast with the photo-aged skin of the superior forehead. The occipital area may also be involved (15–30.4%) [3,13,56]. In men, the loss of sideburns may be the only sign of the disease [112]. The alopecic area appears as a shiny, atrophic, and pale band of incomplete hair loss [113]. 

When the original hairline is missing, the maneuver of cocking both eyebrows may help to find it: a sharp muscular demarcation is noted between the forehead and the scalp [114]. Wood’s light examination may also help to define the missing hairline [115].

**Figure 2:**
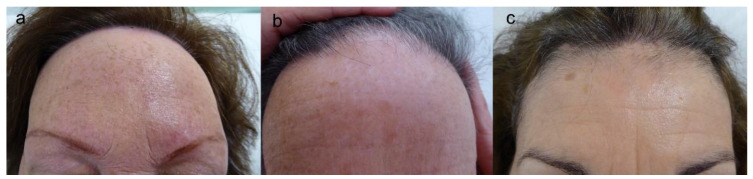
**(a**) Pattern I: linear and uniform hairline recession, without loss of hair density behind the new hairline. (**b**) Patter II: diffuse alopecia behind the frontal hairline with loss of hair density behind. (**c**) Pattern III: unaffected primitive frontal hairline followed by an alopecic band, forming the pseudo “fringe sign”. Note the absence of eyebrow alopecia.

**Figure 3:**
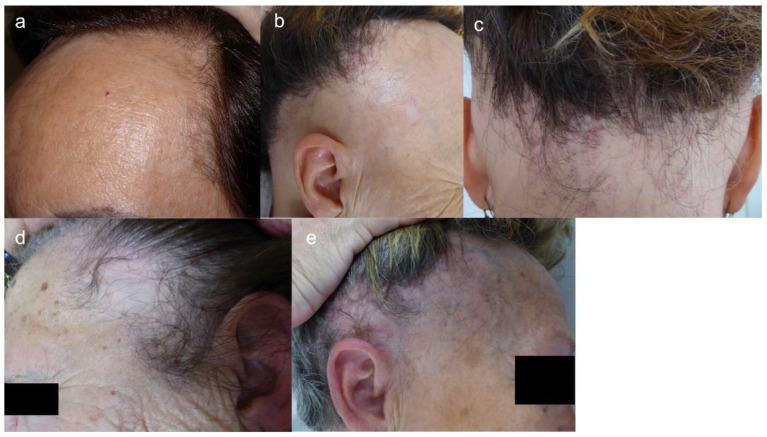
(**a**) Recession of both fronto-temporal hairlines, mimicking male AGA (AGA-like pattern). (**b**,**c**) Recession of the whole hairline, from frontal to occipital (ophiasis-like pattern). (**d**) Oval alopecic patches in the temporal region, sparing a thin band of temporal hairline (cockade-like pattern). (**e**) Recession of temporal hairline extending upwards to the parietal scalp (upsilon pattern). In this patient, the frontal hairline is also affected, but not the occipital area, and neither is the retroauricular region.

The hairline recession is usually bilateral and symmetric [12], but asymmetric forms have also been described. Advanced cases can lead to a “clown alopecic pattern”, with total hair loss in the frontoparietal area [12]. Three clinical patterns of FFA, established according to frontal hairline recession, have been described (Table 2, Figure 2) [116]. Unusual patterns have also been reported (Table 2, Figure 3) [117,118].

The presence of isolated hairs in the original hairline is a helpful diagnostic clue (lonely hair sign) [119]. Sometimes, the unusual retention of hair along the frontotemporal rim produced a pseudo “fringe sign” [120]. Loss of the vellus and intermediate hairs along the primitive hairline gives an appearance of a doll hairline [121].

Partial or total eyebrow alopecia is noted in around 63–83% of patients [2,12,13,14,122]. Eyebrow alopecia can start as hair loss in the external third of the eyebrow or as a diffuse thinning, and can occur either before (more than a third of cases) or after the onset of the hairline recession, without clinical inflammation (although diffuse erythema and pruritus may be associated with eyebrow loss) [12,14,66,123,124]. Sometimes, eyebrow alopecia may be the only sign of FFA [123]. Eyelash alopecia can also be noted (3–14%) [2,3,13]. In men, the beard can also be affected (8–55%) [3,18,74,125].

Clinically non-inflammatory peripheral hair loss, that is, axillary, pubic, and mainly extremity hair loss, is found in 22–77% of patients with FFA [13,14,66,126], generally occurring before hair scalp loss [127]. Interestingly, a patient with FFA with upper limb alopecia developed hypertrichosis in the forearm after removal of a plaster cast [128]. 

Facial papules due to vellus hair involvement are another common finding (6–37%) [3,14,129,130]. They are distributed over the facial skin and are more visible over the temples, although they may also appear on the cheeks or chin [131]. No inflammatory signs are associated with these papules (although they might also be erythematous in patients with light phototypes) [132], and facial vellus tend to be less or absent [133]. Sometimes, these papules may be keratosis pilaris-like, with keratin-filled dilated infundibula [131]. Papules are more prevalent or can be better noted in younger patients (premenopausal) [68], probably because they appear early in the course of the disease, or because they are more easily visible without wrinkles and solar elastosis [131]. In addition, facial papules may disappear over the years, leaving smoother skin without visible follicular openings [131]. They are more frequent in Hispanics/Latinos, similar to other facial lesions in FFA [68]. Some studies suggest that facial papules, as well as eyelash loss and body hair involvement, are associated with severe forms of FFA [3]. Moreover, some authors consider that facial papules (33–50%) and occipital involvement (33%), AGA (67–83%), and body hair loss (42–83%) may be more frequent in men with FFA than in women [3,74], although they seem to have a lower incidence of eyebrow involvement and hypothyroidism [18,20]. Recently, yellow facial papules have been described [134].

Follicular red dots are also a clinical sign of vellus follicle involvement [135]. They are sometimes associated with follicular keratosis and may be noted in the glabella, forehead, eyebrows, cheeks [14,131,135], or even on the body (hip, chest) [136,137].

Other facial lesions have been identified in FFA, such as a more diffuse erythema, especially over the eyebrows and cheeks, or a generalized erythema on the facial skin and neck, adopting a reticular pattern [131]. The erythema tends to disappear progressively, and sometimes lentiginous blue-grey or brown perifollicular macules may gradually appear. This diffuse erythema may be related to the higher prevalence of rosacea described in patients with FFA, especially the erythematotelangiectatic form [19].

LPPigm may be associated to FFA, mainly in dark skin types, such as Hispanic/Latino and black-skinned patients (44–54%) [24,69], as well as Asians [24]. It appears as brown to grey macular pigmentation mostly on the face and neck, but also in flexural areas [15,68,138]. LPPigm seems to be more frequent in premenopausal women, and precedes the onset of FFA in many cases [70].

Depression of the frontal veins has been described as another clinical sign of FFA [139], probably due to atrophy of the overlying skin of the forehead, and has been associated with a worse initial hairline recession and initial and final eyebrow involvement [116]. This finding appears independently of the use of topical corticosteroids, although their use may worsen the condition.

Other signs described for FFA are the presence of increased pre-auricular lines in patients with FFA [140] and increased sweating of the scalp [40]. Follicular re-pigmentation of the white/grey hair in the frontal, temporal, and occipital hairline in patients with FFA has also been reported [141,142].

Regarding symptoms, some patients have pruritus (35–53%) and/or trichodinia (20–25%) in the hairline [3,68], which seem to be less frequent in the occipital area compared to the frontal hairline [143]. Moreover, older patients with FFA seem to be more likely to have anxiety or depression [144].

### 4.2. Clinical Course and Prognostic Factors

FFA is usually insidious, but can be rapidly progressive, and may also remain static for periods of time [13] or become stabilized spontaneously after several years of evolution [2,3]. However, the level of progression before stabilization is unpredictable. Without treatment, the hair loss per year, measured by the distance of recession of the hairline, may range from 0.2 to 2.1 cm [3]. In the early disease, eyebrow regrowth may be achieved with some local treatments. The patient’s age and age at disease onset are both predictors of FFA severity, with higher age and age at onset being related to more severe forms [109]. A lower educational level might also be associated with severe forms of the disease [109,145]. Higher body mass index has been found to be associated with severe forms [109], as well as with the presence of rosacea in patients with FFA [19]. Moreover, patients with more severe FFA seem to be more likely to have rosacea [19]. Regarding clinical patterns, pattern III is associated with the best prognosis and pattern II with the worst, whereas pattern I has an intermediate prognosis [116]. 

## 5. Trichoscopy

**Figure 4:**
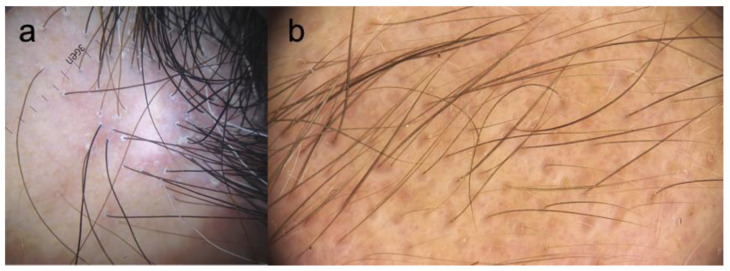
(**a**) Frontal hairline: perifollicular erythema and hyperkeratosis, follicles with one hair shaft, white patches, and loss of follicular openings. (**b**) Eyebrows: partial alopecia with red follicular dots.

Perifollicular erythema and follicular hyperkeratosis, along with the loss of follicular openings in the affected hairline, are the main trichoscopic findings in FFA (Figure 4a) [22]. The presence of follicular ostia with only one hair shaft is another frequent feature. The background in FFA is ivory-white [146]. Perifollicular hyperpigmentation, as well as pinpoint white dots in the alopecic band, are characteristics of darker-skinned patients with FFA [15,16,147]. Black dots, broken hairs, pili torti, and branching capillaries may also be seen, as well white patches in advanced disease [148].

Yellow dots may also be found in FFA, and may be an early feature associated with follicles with a potential for regrowth [149]; therefore, they are more frequent in mild cases [150]. 

In the temporal area, where follicular hyperkeratosis and perifollicular erythema are rarely seen [116,151], a characteristic finding is that most of the hair shafts show transparent proximal hair emergence [151].

The presence of solitary terminal hairs at the site of the original hairline [119] and the absence of vellus hair in the frontotemporal hairline [152] are very helpful clinical clues to the diagnosis of FFA, and also help to rule out other differential diagnosis. Loss of vellus hair in the frontal hairline is the most common trichoscopic sign in mild cases of FFA, although it may be partially or totally preserved in some of them [150].

Although perifollicular erythema has been considered as a marker of FFA activity [153] and many patients with a receding hairline have persistent inflammatory signs (perifollicular erythema and scaling), there is growing recognition that these inflammatory signs can persist in patients despite there being no progression in hairline recession [13,14], and others may have hair loss progression without inflammatory signs [154]. Moreover, the presence of peripilar erythema has been correlated with the coexistence of rosacea [19]. On the other hand, patients with pubic hair loss presented more cicatricial white patches, and those have been associated with the severity of FFA [155]. 

In the eyebrow area, the presence of a few black dots and dystrophic hairs may suggest the diagnosis of FFA [123]. Red or grey dots (Figure 4b) may indicate a favorable prognostic factor for local regrowth, while loss of follicular openings and pinpoint dots within whitish areas are seen in advanced disease [124]. Eyebrow regrowth in distinct directions and pili torti may also be noted [123,124,156]. Tapered hair, broken hair, and yellow dots may be observed in FFA eyebrows, but less frequently than in AA [124]. 

Four dermoscopic patterns of LPPigm in patients with FFA have been described: pseudo-network, speckled blue-grey dots, dotted pattern, and blue-grey dots arranged in circles [138]. 

A recent study found that telangiectasias, red dots, follicular plugs, and perifollicular erythema are more frequent in phototypes I–III, while peripilar hyperpigmentation, black dots, dystrophic hairs, short thin hair/vellus, peripilar casts, and broken hairs are more frequent in phototypes IV–VI [157]. 

Vascular structures (arborizing vessels and extravasated hemorrhages) become more common when there is chronic use of topical corticosteroids, whereas perifollicular erythema and the peripilar cast become less visible [158].

Ultraviolet light-enhanced trichoscopy may be helpful to predict the efficacy of local treatment, so that positive fluorescence (“starry night sky sign” pattern), which is due to the presence of Propionibacterium acnes, may be a sign of a still-preserved hair follicle [159].

## 6. Clinical Classification and Severity Scores

A five-grade classification to assess the clinical severity of FFA has been proposed: I (<1 cm), II (1–2.99 cm), III (3–4.99 cm), IV (5–6.99 cm), and V (>7 cm, also called “clown alopecia”). This size is obtained by measuring the area of cicatricial skin produced by the recession of the frontal and temporal hairline and using the largest measurement to define the grade of severity [3]. 

Currently, two validated scoring systems for FFA assessment exist: the FFASI (FFA Severity Index), which gives scores for hairline recession, inflammatory band, non-scalp loss, and associated features [160], and the FFASS (FFA severity score), which also includes signs of local inflammation and patients’ symptoms [161].

## 7. Laboratory

Blood analysis, including hemogram, general biochemical, liver function, thyroid function, antinuclear antibodies, and sex hormones, is usually normal [12]. Low levels of positivity for antinuclear antibodies have been found in some patients with FFA [2,12]. Therefore, blood tests seem to be unnecessary in FFA, except for rejecting thyroid disorders.

## 8. Image Techniques

Optical coherence tomography in FFA has shown increased epidermal thickness in the inflammatory hairline and decreased thickness in the alopecic band, as well as a lower vascular flow in the alopecic band compared to the inflammatory scalp in the superficial dermis, but increased flow in the deeper plexus [162]. 

Reflectance confocal microscopy in LPP and FFA allows visualization of the major key diagnostic features, such as infundibular hyperkeratosis, perifollicular lichenoid inflammatory infiltrate, and extensive perifollicular fibrosis [163].

Regarding sonography, FFA patients have a higher vessel diameter and flow in the hairline implantation area in comparison with a control group, which may be explained by the presence of active inflammation [164]. Interestingly, the vessel diameter has been demonstrated to be higher in the healthy scalp area in FFA patients than in a control group, which may reflect the presence of subclinical inflammation in the still unaffected areas. The presence of branched vessels has been related to a higher significant flow in the hairline area in those patients, independently of the use of topical corticosteroids, so these vessels may be the reflex of active inflammation. 

## 9. Histopathology

Histological findings in FFA seem to be indistinguishable from LPP, according to some authors [165]. FFA is characterized by a lichenoid lymphocytic infiltrate around the upper follicle, i.e., isthmus and infundibulum, including the bulge area, as well as concentric perifollicular lamellar fibrosis [1]. A strong correlation between the severity of the peripilar cast and the degree of lymphocytic infiltration has been identified [166]. It is thought that the destruction of the external root sheath at the level of the isthmus is responsible for irreversible alopecia [167]. The lower part of the follicle usually remains spared. A reduction in the number of hair follicles is a consequent finding: a mean of seven terminal hair follicles have been seen per 4 mm punch biopsy in FFA, whereas “normally” around thirty terminal and five vellus hair follicles are seen in Caucasians, and eighteen terminal and three vellus ones in Afro-Caribbeans [66]. 

The loss of sebaceous glands is an early finding in FFA [120]. Indeed, in eyebrow samples, sebaceous gland preservation may be the pathological correlation for the reversibility of eyebrow loss [168]. In early cases, the inflammatory involvement of the vellus follicles and atrophy of the sebaceous glands are the histological clues, not including perifollicular fibrosis [169].

Vacuolar degeneration of the basal layer, keratinocyte necrosis, and replacement of the pilosebaceous units by fibrous tracts, along with loss of elastin fibers, are other FFA signs [170]. Advanced cases may only reveal fibrous tracts and the absence of hair follicles, without any inflammatory infiltrate, similar to other cicatricial alopecias in their later stage [12]. Dilated eccrine glands have been identified in a patient with FFA, together with increased scalp sweating [40].

Initially, it was considered that intermediate and vellus-like follicles were more commonly affected than terminal follicles by the lymphocytic inflammatory infiltrate and perifollicular fibrosis [5]. Nowadays, it is accepted that terminal follicles are involved in the same way as the others [167]. The follicular triad has been described in early FFA, and describes the simultaneous involvement of follicles of different types (terminal, intermediate, and vellus) and in a different stage of the cycle (anagen, catagen, and telogen) [171]. 

Regarding the composition of the inflammatory infiltrate in FFA, this is characterized by an increase in the percentage of CD8+ T cells [37,172], with a reversal of the typical CD4:CD8 ratio (which is approximately 2:1). However, this ratio is increased (>3:1) in uninvolved follicles in FFA, which may be because of the migration of CD8+ T cells from uninvolved areas to involved ones [170]. A significant increase in perifollicular and interfollicular Langerhans cells has also been described in FFA. Plasmocytoid dendritic cells, the most potent I IFN producers, are increased in LPP and FFA, mainly confined to the upper dermis surrounding the hair infundibulum [173]. Moreover, a reduced number of CD1a+ and CD209+ dendritic cells in the perifollicular mesenchyme adjacent to the infundibulum in both LPP and FFA has been described, whereas increased total numbers and degranulation status of perifollicular mast cells have been found in lesional LPP and FFA hair follicles [37].

A lower melanocyte count has been demonstrated in the upper follicle in FFA patients compared to LPP, and is associated with the hypopigmentation observed clinically in the alopecic band in FFA [26,30]. 

A recent study provides data for significant immune dysregulation in FFA, with increased infiltration of CD8+ T cells, CD11c+ dendritic cells, CD69+ and CD103+ TRM, tryptase+ mast cells, and FOXP3+ Tregs, in addition to significant upregulation of Th1 and JAK-STAT pathways [172]. Moreover, K15 and CD200 expression in the hair follicle bulge has revealed some preservation of stem cells in lesional FFA [172].

Few histological abnormalities have been described in clinically unaffected scalp in FFA and LPP, such as infundibular lymphocytic inflammation [174,175,176] and early sebaceous gland atrophy [165]. Perifollicular fibrosis and mucin deposits have also been noted in unaffected scalp [176]. On the other hand, a relative increase of CD4 + FOXP3 + T regulatory cells in the perifollicular infiltrate in both affected and unaffected scalp in FFA has been described [175].

Recently, dermal fat infiltration at the isthmus level and in the arrector pili muscle has been observed in FFA samples [177]. Interestingly, the arrector pili muscle is thought to play an important role in protecting the stem cells in the bulge area. Moreover, a dermal displacement of eccrine sweat coils has also been noted in a fair number of patients. 

Biopsy of the facial papules also reveals follicular hyperkeratosis and lichenoid dermatitis involving the infundibular and isthmus portions of the vellus hair follicles [131], or even fibrosis around the vellus hair or complete follicular destruction [130,133]. Interestingly, sebaceous glands are present in the majority of cases, different from the picture one might expect in scalp samples [178]. Indeed, in most cases, prominent sebaceous glands with dilated ducts are seen. The destruction of elastic fibers may be responsible for the “pop-out” of sebaceous glands and the formation of the yellow facial papules [134]. A lymphocytic folliculitis without perifollicular lamellar fibrosis has been detected in some cases of limb biopsies, similar to frontal scalp biopsies in early FFA [179]. The histopathology of LPPigm is characterized by epidermal atrophy, mild vacuolar dermatitis, sparse perivascular lymphocytic infiltrate (in early phases), and pigment incontinence, and can share the pattern of lichenoid folliculitis, also observed in biopsies from the scalp, eyebrows, limbs, and facial lesions of patients with FFA [180].

With regards to eyelashes, small and narrow bulbs, irregular caliber, and irregular pigment distribution have been observed. Demodex folliculorum infestation was noted in a patient with mild FFA and eyelash loss, which suggests that it might accelerate autoimmune inflammation and produce premature eyelash alopecia [181].

## 10. Are LPP and FFA the Same Disease?

Whether FFA is a variant of LPP or a different entity is still unclear [2,3]. Clinically, LPP is usually associated with multifocal areas of scarring alopecia that may coalesce to produce large alopecic areas [3]. The most typical locations are the vertex and parietal areas, although it may extend throughout the scalp in a patchy manner [167]. Moreover, LPP is associated with LP at other sites more often than FFA is [167]. Regarding trichoscopic signs, perifollicular hyperkeratosis in LPP is more intense than in FFA, and the background is typically milky-red [146]. 

FFA and LPP share main histological features. However, a few differences have been found between LPP and FFA (Table 3), which makes it more suitable to consider FFA as a specific type of lymphocytic cicatricial alopecia rather than a variant of LPP [167]. The inflammatory infiltrate in FFA is usually milder than in LPP [167,182]. According to one study, most FFA patients exhibit the maximum degree of inflammation at the isthmus, but a significant number of patients with FFA may have inflammation extending below the isthmus, or even fibrosis, in comparison with LPP [183]; however, this has not been supported by other studies [172]. Moreover, the damage to the basal layer tends to be subtler in FFA than in LPP [167]. The presence of a superficial perivascular lymphohistiocytic inflammatory infiltrate is common in LPP, but not in FFA [167]. Eosinophilic necrosis of keratinocytes of the external root sheath is prominent in FFA, especially at the isthmus, whereas it is not as marked in LPP and, if present, is located at the lower follicle [167]. A foreign body reaction following follicular destruction is usually more intense and frequent in FFA than in LPP [167]. Moreover, the interfollicular epidermis is commonly spared in FFA, but it is affected in 50% of cases of LPP [167]. Furthermore, concentric lamellar fibroplasia seems to be more frequently present in LPP than in FFA, while the presence of terminal catagen-telogen hairs is more frequently found in FFA [182]. The presence of direct immunofluorescence deposits is more frequent in LPP [167,184]; in FFA, it is usually negative, although IgM deposits over the basement membrane and cytoid bodies in the papillary dermis have been described [133,185]. Moreover, the epidermis is thinner in FFA than in LPP [30]; this reduction in the epidermal thickness (and also dermal) has been noted in the alopecic band of FFA naïve patients [174]. A recent study has found that macrophages exist in different functional phenotypes in LPP and FFA; CD86 is downregulated in LPP compared with FFA, whereas CD163 is increased in LPP and decreased in FFA [37]. 

A lower melanocyte count has been demonstrated in the upper follicle in FFA patients compared to LPP [26].

## 11. Diagnosis

Diagnostic criteria for FFA are referred to in Table 4 [186,187].

## 12. Differential Diagnosis

The ophiasis pattern of AA, which affects the margins of the scalp, may masquerade as FFA. In addition, AA may produce eyebrow alopecia, sometimes as an isolated finding. However, perifollicular erythema and hyperkeratosis are absent in AA, whereas yellow dots, dystrophic and broken hairs, black dots, exclamation mark hairs, tapered hairs, and regrowing hairs are common features [146]. 

Traction alopecia may resemble FFA. The clinical background, with a history of use of tight hairstyles and the absence of typical trichoscopic signs of FFA, may be useful. Moreover, traction alopecia is not associated with eyebrow hair loss.

Other scarring alopecias, such as LPP, discoid lupus erythematosus, and pseudopelade of Brocq, tend to produce multifocal alopecic areas. 

A familial high frontal hairline should also be discarded.

AGA with male pattern may also be considered, especially when the frontal or temporal hairline is receded. However, hair miniaturization (with an increased proportion of thin and vellus hair) and anisotrichia [146] are not trichoscopic signs in FFA.

## 13. Treatment

Almost all information about treatment in FFA is based on retrospective cohort studies and cases reports [12,13,188]. The aim of the treatment is to alleviate symptoms and signs and arrest the progression of the hair loss, since hair regrowth is not possible once destruction of the follicles has happened.

### 13.1. Local Treatments

Topical corticoids are recommended, especially in the early inflammatory stage, but relapse occurs upon their discontinuation [12,188]. Potent topical steroids and calcineurin inhibitors reduce inflammation, but without any clear benefit in slowing the alopecia [11,13,189], although disease stabilization with a combination of both treatments has been published [190]. However, a study including 92 FFA patients revealed that patients treated with 0.3% tacrolimus were more likely to stabilize in three months compared to those treated with clobetasol/betamethasone [191]. 

With regards to intralesional corticosteroids, 20 mg/mL of triamcinolone acetonide used in the hairline (every 3–6 months) may obtain hair regrowth in some patients [11]; 34% of patients showing improvement, 49% stabilization, and 5% worsening are the outcomes found in a cohort of 130 patients [3]. It is also a useful treatment in eyebrow alopecia, where 10 mg/mL (or even more diluted) every three months, may obtain hair regrowth, especially in cases of partial eyebrow loss [11,122]. Moreover, it seems to be uncommon for patients with FFA and with eyebrow alopecia to experience eyebrow regrowth with systemic therapy alone [122]. 

Topical minoxidil has not shown clinical improvement in the slowing down of the progression of the alopecia [12].

Bimatoprost 0.03% eye drops, a prostaglandin analogue, may be used for eyebrow loss; a study involving three patients who applied it twice daily showed regrowth in two of them after nine months of treatment [192]. It may also be an option in eyelash loss.

Some authors referred to the fact that excimer laser may be effective in reducing inflammation and peripilar casts in patients with active disease [193]. Application of superluminescent diodes as an adjuvant therapy in patients with FFA/LPP showed a decrease in subjective symptoms and perifollicular hyperkeratosis, and even an increased number of thick hairs within the treated area [194].

One patient of recalcitrant FFA treated with platelet-rich plasma (PRP) (0.1 mL/cm^2^, five treatments with a one-month interval) injected into the frontotemporal hairline and eyebrows showed improvement in trichoscopic signs and no further hair loss after five months [195].

### 13.2. Systemic Treatments

Oral prednisone (0.5–1 mg/kg/day, three to eighteen months) has been shown to produce a stoppage of hairline recession in almost 43% patients, but with a relapse when the treatment is stopped [12]. Intramuscular triamcinolone acetonide (40 mg every three weeks) has also been used, but with no therapeutic effect observed [5].

A study including 36 patients revealed an improvement in symptoms and signs of FFA in 73% of patients treated with hydroxychloroquine, though most were partial responses. The maximal benefits were seen within the first six months of therapy. On the other hand, 60% of them produced a response to mycophenolate mofetil, though mainly partial ones [17]. Other reports did not find any consistent benefit with the use of hydroxychloroquine [13].

A report including 102 patients treated with oral finasteride (2.5–5 mg/day), which inhibits the isoenzime type II of 5-alpha reductase, displayed that 47% of patients showed improvement (regrowth in the hairline) and 53% showed a stabilization of the alopecia [3]. There is even one report of a woman with FFA who experienced frontal hair regrowth and reversal of cutaneous atrophy within three to twelve months of treatment with oral finasteride [196]. Subsequently, favorable outcomes have been described with oral dutasteride, which is about three times as potent as finasteride at inhibiting type II 5-alpha reductase and more than 100 times as effective at inhibiting type I [188]. In 18 patients with FFA treated with oral dutasteride (0.5 mg/week), 44% of patients displayed improvement and 56% of them showed a stabilization of the alopecia [3]. Moreover, a report combining oral dutasteride with topical pimecrolimus revealed a stoppage in hairline recession along with hair regrowth in eyebrows and axillae [188,189]. Other authors have found a stabilization of hair loss in 70% of FFA patients treated with dutasteride and in 33% of patients treated with finasteride [129]. Therefore, oral 5-alpha reductase inhibitors seem to be the most effective therapy in FFA patients, as all patients experienced at least stabilization [3]. This oral therapy should be accompanied by intralesional corticosteroid infiltration in the hairline when inflammatory signs are present [3]. 

A retrospective analysis of 54 women with FFA treated with oral isotreinoin (20 mg/day), acitretin (20 mg/day), or finasteride (5 mg/day) showed a stoppage of progression in 76%, 73%, and 43% of patients, respectively [197]. Alitretinoin has also been used in a woman with FFA, who showed improvement after one month with 30 mg/day [198].

Recently, a study including 224 FFA patients has compared the effectiveness of oral dutasteride against other systemic treatments (finasteride, hydroxychloroquine, doxycycline, isotretinoin) and with a group receiving no systemic therapy (just topical minoxidil 5% and clobetasol propionate 0.05% solution) [199]. Authors found significant differences in the percentage of stabilized patients after twelve months of therapy between patients treated with dutasteride versus the other groups, with a stabilization rate of 61.5–64.2%. The response was dose-dependent, and the most effective dose was five to seven capsules of dutasteride (0.5 mg) per week.

Regarding facial papules, an improvement of the facial skin surface and regrowth of vellus after six months of treatment with oral prednisone and antimalarials have been reported [133]. A favorable outcome has also been published with oral isotretinoin (10 mg/day) in two to four months [134,182]. 

The only randomized controlled trial is in regard to the use of oral isotretinoin (initially 20 mg/day, then 20 mg every other day after one month) combined with topical clobetasol (0.05%) and tacrolimus (0.1%) compared to topical treatment alone in FFA [200]. Authors found improvement of facial papules, no deterioration of the FFASI variables, and improvement of erythema and perifollicular keratosis in the frontal line in the treatment group after six months.

Other treatments, such as griseofulvin, azathioprine, or tetracyclines, have shown no efficacy or inconsistent outcomes so far [1,2,5,11,17,129]. Few patients have achieved stabilization of their FFA during treatment with methotrexate [129,201].

Pioglitazone hydrochloride (15 mg/day), an oral PPAR-γ agonist, showed improvement of itching and a decrease in the inflammatory infiltrate in a patient with LPP, but no remission [202]; however, studies with more patients have shown a negative outcome in most patients [203]. No successful results have been observed in FFA patients [129].

Oral minoxidil has been demonstrated to improve the background hair thickness in LPP, especially in patients with diffuse LPP; however, the report excluded patients with FFA [204]. Further studies regarding its efficacy in FFA are needed.

With regards to biological therapies, a study including 10 patients with recalcitrant LPP and FFA (2/10) who were treated with oral tofacitinib, a pan-JAK inhibitor, 10–15 mg/day from two to nineteen months, showed a clinical response in 80% of patients and clinical improvement in both FFA patients [205]. One woman who had refractory FFA and LPP showed improvement after around four to thirteen months of treatment with tildrakizumab (100 mg subcutaneously at week zero, four and subsequently twelve weekly), an anti p19 IL23 monoclonal antibody [206]. One case of a woman who was receiving adalimumab for hidradenitis suppurativa and rheumatoid arthritis and experienced hair regrowth in the area affected by LPP has been reported [207].

### 13.3. Hair Transplant

A minimum of one to five years without activity is recommended before hair transplantation is used in scarring alopecias [208,209]. Most FFA patients who underwent hair transplantation lost the hair grafts in around four years, suggesting that FFA displays recipient dominance [209,210]. Similar results have been observed in eyebrow transplantation [211]. A recent study has shown a decrease in graft survival over time, independently of the period of time since clinical remission, with a graft survival rate lower than 60% after five years [212]. Therefore, a hair transplant should only be offered to selected patients with FFA to improve small areas and after first discussing with the patient the long-term survival rate of the grafts.

**Figure 5:**
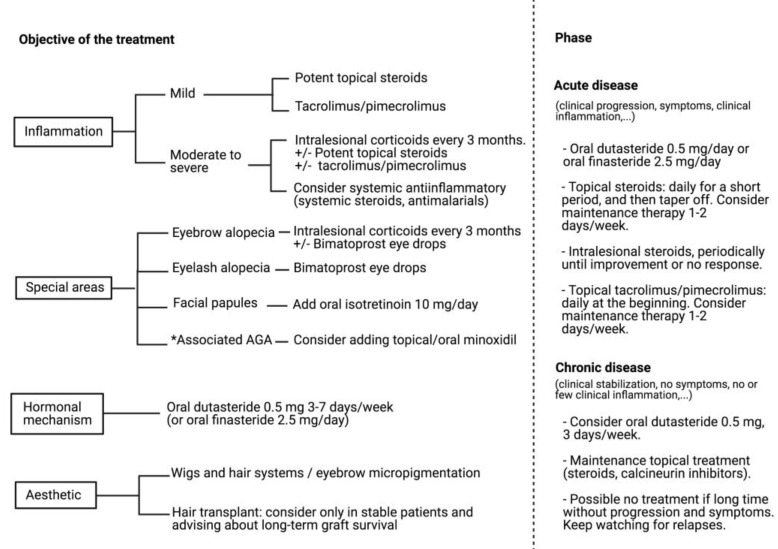
Algorithm of treatment. * Some patients with FFA may associate AGA; in these cases, adding minoxidil may provide an additional therapeutic benefit. Created with BioRender.com (accessed on 1 March 2021).

According the published data, a simplified algorithm of treatment is represented in Figure 5.

## 14. Conclusions

FFA prevalence has increased during the last few years, and so has the interest of the medical community regarding its characterization, pathogenesis, and treatment. However, most studies are observational reports, so further investigations and clinical trials are needed to clarify important issues, such as the possible responsible trigger. In that way, further studies about hair and skin cosmetic routines in patients with FFA may be an interesting research prospect. Moreover, the study of the existence of environmental factors that may explain differences in the prevalence of FFA in different geographic areas could contribute to a better understanding of FFA. The precise knowledge of its ethology and pathogenic mechanisms may expose specific therapeutic targets. Although the use of 5-alpha reductase inhibitors has permitted the stabilization of a considerable number of patients, research for new treatments, such as oral minoxidil or biological therapies, is still lacking. 

## Figures and Tables

**Table 1:** Autoimmune diseases described concomitantly with FFA.

Autoimmune Associated Diseases
Thyroid disorders: Hypothyroidism (8–44.6%) Hashimoto thyroiditis (8.1%) Graves disease (1.4%)
Lichen planus (1.7–18.2%): Cutaneous (3–6.5%) Mucosal (3–16.7%) Pilaris (0.8–25.3%)
Psoriasis (7.4%)
Vitiligo (0.6–5.6%)
Inflammatory bowel disease (5.4%)
Lichen sclerosus (0.3–5.4%)
Sjögren syndrome (1.7–4.1%)
Discoid cutaneous lupus erythematosus (-)
Systemic lupus erythematosus (3.4%)
Coeliac disease (1.5–2.0%)
Pernicious anaemia (1.2–1.7%)
Alopecia areata (0.6–1.7%)
Scleroderma (1.4%)
Rheumatoid arthritis (1.4%)
Polymyalgia rheumatic (-)
Primary biliary cirrhosis (-)
Mucous membrane pemphigoid (-)

(-) Data from isolated reports.

**Table 2:** Typical and unusual patterns of FFA.

Pattern Name	Clinical Description
**Typical Patterns**	
Pattern I (linear)	Uniform band of frontal hairline recession in the absence of loss of hair density behind the hairline
Pattern II (diffuse)	Diffuse or zigzag band-like alopecia affecting the frontal hairline with significant loss of hair density behind the hairline (at least a 50% decrease in normal hair density) with a compatible trichoscopy.
Pattern III (pseudo-fringe-sign)	Unaffected primitive frontal or temporal hairline forming the pseudo “fringe sign.”
**Unusual Patterns**	
AGA-like pattern	Symmetric recession of frontotemporal hairlines, with sparing of the paramedian frontal hairline (mimicking male pattern AGA).
Ophiasis-like pattern	Continuous involvement of the hairline from frontal to occipital regions.
Cockade-like pattern	Presence of oval patches of alopecia in the temporal regions, with sparing of a band of temporal hairlines.
Upsilon pattern	Band-like pattern along the frontotemporal scalp extending into two symmetrical triangles along the parietal scalp.

AGA: androgenetic alopecia.

**Table 3:** Histopathological differences between FFA and LPP.

Histological Features	FFA	LPP
Inflammatory infiltrate degree	+	++
Inflammation/fibrosis below the isthmus	++/−	+/−
Basal layer damage degree	+	++
Superficial perivascular infiltrate	+/−	++
Keratinocyte necrosis in the external root sheath	++	+
Foreign body reaction	++	+/−
Involvement of interfollicular epidermis	−	++
Concentric lamellar fibroplasia	+	++
Presence of terminal catagen-telogen hairs	++	+/−
Direct immunofluorescence deposits	+/−	+
Epidermal thickness reduction	++	+
Macrophage polarization	+CD86, −CD163	−CD86, +CD163
Lower melanocyte count in the upper follicle	+	−

FFA: Frontal fibrosing alopecia. LPP: lichen planopilaris. (−) Absence, (+) Presence. (++) instead of (+) indicate a higher degree or intensity compared to the other (FFA vs LPP).

**Table 4:** Diagnostic criteria for FFA.

Major Criteria	Minor Criteria
1. Cicatricial alopecia of the frontal, temporal, or frontotemporal scalp, in the absence of follicular keratotic papules on the body.	1. Typical trichoscopic features (perifollicular erythema and/or follicular hyperkeratosis, lonely hair sign).
2. Diffuse bilateral eyebrow alopecia.	2. Histopathological features of FFA and LPP.
	3. Involvement (hair loss or perifollicular erythema) of additional FFA sites (occipital area, facial hair, sideburns, or body hair).
	4. Non-inflammatory facial papules.
	5. Preceding or concurrent symptoms (pruritus or pain) at the areas of involvement.

The diagnosis of FFA requires two major criteria or one major criterion and two minor criteria.
